# Huntingtin gene repeat size variations affect risk of lifetime depression

**DOI:** 10.1038/s41398-017-0042-1

**Published:** 2017-12-11

**Authors:** Sarah L. Gardiner, Martine J. van Belzen, Merel W. Boogaard, Willeke M. C. van Roon-Mom, Maarten P. Rozing, Albert M. van Hemert, Johannes H. Smit, Aartjan T. F. Beekman, Gerard van Grootheest, Robert A. Schoevers, Richard C. Oude Voshaar, Raymund A. C. Roos, Hannie C. Comijs, Brenda W. J. H. Penninx, Roos C. van der Mast, N. Ahmad Aziz

**Affiliations:** 1 Departments of Neurology, Leiden University Medical Centre, Leiden, The Netherlands; 2 Departments of Human Genetics, Leiden University Medical Centre, Leiden, The Netherlands; 3 Departments of Clinical Genetics, and Leiden University Medical Centre, Leiden, The Netherlands; 4 Department of Public Health, Section of Social Medicine, University of Copenhagen, Copenhagen, Denmark; 5 Departments of Psychiatry, Leiden University Medical Centre, Leiden, The Netherlands; 6 Department of Psychiatry, and EMGO Institute for Health and Care Research and Neuroscience Campus Amsterdam, VU University Medical Center/GGZ inGeest, Amsterdam, The Netherlands; 7 Department of Psychiatry, University Medical Centre Groningen, Groningen, The Netherlands; 8 Department of Psychiatry, Collaborative Antwerp Psychiatric Research Institute (CAPRI), University of Antwerp, Antwerp, Belgium; 9 Department of Neurodegenerative Disease, UCL Huntington’s Disease Centre, University College London Institute of Neurology, London, United Kingdom

## Abstract

Huntington disease (HD) is a severe neuropsychiatric disorder caused by a cytosine-adenine-guanine (CAG) repeat expansion in the *HTT* gene. Although HD is frequently complicated by depression, it is still unknown to what extent common *HTT* CAG repeat size variations in the normal range could affect depression risk in the general population. Using binary logistic regression, we assessed the association between *HTT* CAG repeat size and depression risk in two well-characterized Dutch cohorts─the Netherlands Study of Depression and Anxiety and the Netherlands Study of Depression in Older Persons─including 2165 depressed and 1058 non-depressed persons. In both cohorts, separately as well as combined, there was a significant non-linear association between the risk of lifetime depression and *HTT* CAG repeat size in which both relatively short and relatively large alleles were associated with an increased risk of depression (*β* = −0.292 and *β* = 0.006 for the linear and the quadratic term, respectively; both *P* < 0.01 after adjustment for the effects of sex, age, and education level). The odds of lifetime depression were lowest in persons with a *HTT* CAG repeat size of 21 (odds ratio: 0.71, 95% confidence interval: 0.52 to 0.98) compared to the average odds in the total cohort. In conclusion, lifetime depression risk was higher with both relatively short and relatively large *HTT* CAG repeat sizes in the normal range. Our study provides important proof-of-principle that repeat polymorphisms can act as hitherto unappreciated but complex genetic modifiers of depression.

## Introduction

Depression is one of the most common psychiatric disorders with a lifetime prevalence of about 15%^1^. Depression is accompanied by substantial morbidity and mortality and, according to the World Health Organization, it is currently the leading cause of disability worldwide with an estimated 350 million people affected globally^2^. In order to devise more effective therapeutic and preventive strategies, a better understanding of its pathogenesis is crucial. A vital step in elucidating the pathogenesis of depression is to gain more insight into its underlying genetic determinants. However, despite an estimated heritability of between 30 and 50 percent of major depression, genome-wide association studies (GWAS) have only had limited success^3^. Although recently a number of single-nucleotide polymorphisms (SNPs) contributing to risk of depression were identified in GWAS involving tens of thousands of subjects, the effect sizes were very small and the inevitably large group sizes harbor risks of bias due to both phenotypic as well as genetic heterogeneity^4,5^.

One of the causes of the ‘‘missing heritability’’ problem of depression may be that, aside from SNPs, GWAS are unsuitable for detecting the contribution of other important genetic polymorphisms, in particular variably expanded DNA repeat sequences^6–8^. Expansions of simple repeat sequences in genomic DNA have been associated with many hereditary disorders^9^, but their association with common diseases is largely unknown. Among expanded repeat disorders polyglutamine diseases are the most prevalent^9,10^. These diseases are characterized by a trinucleotide (cytosine-adenine-guanine (CAG)) repeat expansion in the translated regions of otherwise unrelated genes, resulting in proteins with expanded polyglutamine domains^10^. The most common polyglutamine disease is Huntington disease (HD), a debilitating neurodegenerative disorder characterized by motor impairment, cognitive decline as well as severe neuropsychiatric disturbances of which depression is one of the most prevalent^11–13^. It is caused by a CAG repeat expansion in the Huntingtin (*HTT*) gene^14^. *HTT* alleles containing 40 or more CAG repeats lead invariably to HD, whereas alleles with 36 to 39 repeats have incomplete penetrance. Larger mutant CAG repeat size is associated with lower age-of-onset and faster disease progression^15–17^. Interestingly, mice transgenic for the HD gene also exhibit depression-like behavior indicating that CAG repeat size variations in this gene directly affect neuronal substrates underlying mood regulation^18–20^.

Recently, it was shown that subjects with *HTT* alleles containing CAG repeat lengths in the upper normal range (i.e., the intermediate range with 27–35 repeats) experienced significantly more depressive symptoms, including apathy and suicidal ideation, compared to control subjects^21–23^. Moreover, a case–control study in patients with major depression estimated the prevalence of incompletely penetrant *HTT* alleles (36 to 39 CAG repeats) at about 3 in 1000, whereas alleles in this range were absent in the control group^24^. However, it is still unknown to what extent more common length variations in the CAG repeat tract of the *HTT* gene, ranging from 6 to 35 trinucleotides, could affect lifetime depression risk. In the current study, we, therefore, aimed to assess the contribution of the whole spectrum of *HTT* CAG repeat length variations to depression susceptibility using data from two well-characterized Dutch cohorts: The Netherlands Study of Depression and Anxiety and the Netherlands Study of Depression in Older Persons^25,26^.

## Methods and materials

### Cohort 1

The Netherlands Study of Depression and Anxiety (NESDA) is a cohort study among 2981 participants aged 18–65 years^25^. Participants were recruited from the general population, general practices, and mental health care institutes. This cohort includes 1973 subjects with a lifetime diagnosis of major depressive disorder (MDD) and/or dysthymia (with MDD diagnosed in 1925 of these participants), 635 subjects with a lifetime anxiety disorder without lifetime depression and 373 healthy controls without (a history of) psychopathology^25^. Diagnoses were made according to the Diagnostic and Statistical Manual of Mental Disorders Fourth Edition (DSM-IV) criteria using the Composite Interview Diagnostic Instrument^27^. Due to insufficient DNA material for some subjects we genotyped a total of 2738 participants (including 1808 depressed and 930 non-depressed persons) from this cohort; however, the lacking DNA samples were missing completely at random.

### Cohort 2

The Netherlands Study of Depression in Older Persons (NESDO) is a cohort study among 510 people aged 60–93 years recruited from both general practices and mental health care institutes^26^. The cohort consists of 378 depressed (including 360 patients with MDD) and 132 persons (without a history of) psychopathology. In NESDO the same methods were applied as in NESDA for diagnosing depression^26^. Due to insufficient DNA material for some subjects we genotyped a total of 485 participants (including 357 depressed and 128 non-depressed persons) from this cohort; however, the lacking DNA samples were missing completely at random.

### Cohort 3

For external validation of our findings we extracted data from a previously published study assessing the role of incompletely penetrant *HTT* alleles in patients with MDD^24^. This study included data on the distribution of *HTT* CAG repeat lengths of 2986 chromosomes from MDD patients and 4007 control chromosomes^24^. Because we could not retrieve the raw data, data extraction was performed from the published article using WebPlotDigitizer (version 3.10)^28^.

### Genotyping

We used a similar genotyping protocol as described previously^29^. In brief, a polymerase chain reaction (PCR) was performed in a TProfessional thermocycler (Biometra, Westburg) with labeled primers flanking the CAG stretch of *HTT* (primer 1: ATGAAGGCCTTCGAGTCCCTCAAGTCCTTC, primer 2: GGCGGTGGCGGCTGTTGCTGCTGC) (Biolegio)^30^. The PCR was performed using 10 ng of genomic DNA, 1x OneTaq mastermix (New England Biolabs, OneTaq Hot start with GC Buffer master mix), 1.5 µL of primer Mix A or B (table A) and Aqua B. Braun water to a final volume of 15 µL. The PCR was performed with 30 cycles of 30 s denaturation at 94 ˚C, 1 min annealing at 60 ˚C and 2 min elongation at 68 ˚C preceded by 5 min of initial denaturation at 94 ˚C and followed by a final 5 min elongation at 68 ˚C. Every PCR included a negative control without genomic DNA, a reference sample of CEPH 1347-02 genomic DNA and two positive control samples with predetermined 17/17 and 23/27 *HTT* CAG repeats. The PCR products were run on either an ABI 3730 or an ABI 3130 automatic DNA sequencer (Applied Biosystems) and analyzed using the GeneMarker software version 2.4.0. All assessments were done with cases and controls randomized on plates and blinding with respect to disease status information.

### Statistical analysis

All data are displayed as means and 95% confidence intervals unless otherwise specified. In order to assess whether *HTT* CAG repeat size is associated with risk of lifetime depression we used binary logistic regression analysis. First, to evaluate the presence of potential interaction effects between the two *HTT* alleles or non-linear effects of *HTT* CAG repeat size variations we used a model with the presence of lifetime depression (i.e., MDD and/or dysthymia) as the dependent variable and *HTT* CAG repeat sizes in both alleles, a quadratic term for each allele as well as a product term of the two alleles as explanatory variables. As in this model only the quadratic term of the longer allele appeared to be significantly associated with depression, we reduced the model to contain only a main and a quadratic term for the longer allele as independent variables. Exclusion of the shorter allele hardly influenced the parameter estimates of the longer allele. Therefore, for the sake of simplicity, from here onwards we will take *HTT* to mean the longer of the two alleles of each individual. We also adjusted the results for the effects of sex, age and education level (coded as ‘‘basic’’, ‘intermediate’ and ‘‘high’’^25,26^) in order to assess whether the effect of *HTT* CAG repeat size is independent of these well-established risk factors for depression. The Nagelkerke *R* ^2^ was used to assess the proportion of variability explained by the predictors in the model^29^. In order to account for potential effects of points with high leverage or influential points all statistical significance tests were based on robust estimators of standard errors. Moreover, in order to analytically assess whether there were any influential points of relevance that might have affected our analyses, we also calculated the Cook’s distance, which indicated no major issues with influential points. Furthermore, in order to assure that the results were not unduly affected in case of deviations from model assumptions and explore associations further, we also applied a non-parametric method by calculating odds ratios for a lifetime diagnosis of depression per *HTT* CAG repeat size (collating alleles with repeat sizes of ≤ 16 or ≥ 27 due to their relatively low frequency) compared to the odds of lifetime depression in the total cohort using the Fisher’s exact test. All tests were two-sided and significance level was set at *P* < 0.05. All analyses were performed in SPSS version 23.0 (IBM SPSS Statistics for Windows, IBM Corp).

## Results

### *HTT* CAG repeat size variations influence risk of lifetime depression

**Fig. 1:**
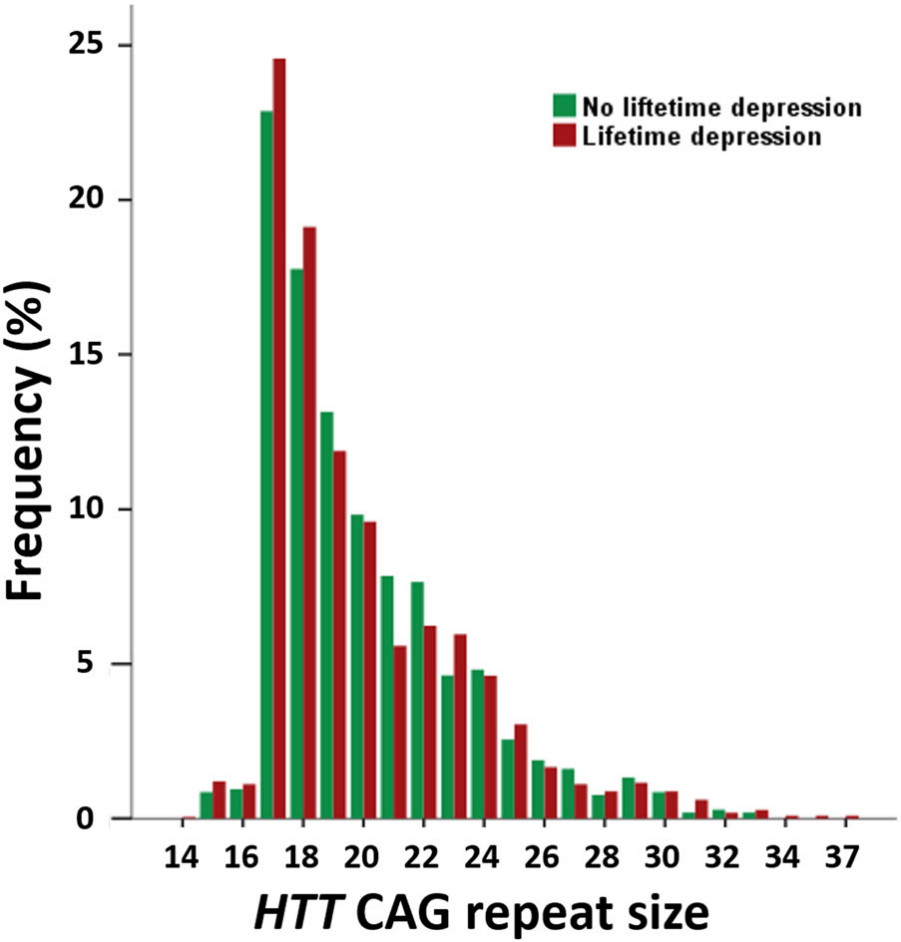
Distribution of *HTT* CAG repeat sizes The figure displays the frequency distribution of the number of CAG repeats in the *HTT* gene in 2165 subjects with and 1058 subjects without a lifetime depression diagnosis, respectively

The distribution of the *HTT* CAG repeat size in the combined cohorts 1 and 2 is displayed in Fig. 1. In cohort 1 there was a significant association between the risk of lifetime depression and *HTT* CAG repeat size in which the association was best represented by a model including a quadratic term for *HTT* CAG repeat size (*β* = −0.239 and *β* = 0.005 for the linear and the quadratic term, respectively; both *P* ≤ 0.044). Adjusting the analysis for the effects of sex, age, and education level hardly changed the results (*β* = −0.249 and *β* = 0.005 for the linear and the quadratic term, respectively; both *P* ≤ 0.040). Similarly, in cohort 2 there was a significant quadratic association between the risk of lifetime depression and *HTT* CAG repeat size (*β* = −0.626 and *P *= 0.054 for the linear term; *β* = 0.015 and *P* = 0.045 for the quadratic term), which became slightly more pronounced after adjustment for the effects of sex, age and education level (*β* = −0.788 and *β* = 0.019 for the linear and the quadratic term, respectively; both *P* ≤ 0.034).

**Fig. 2:**
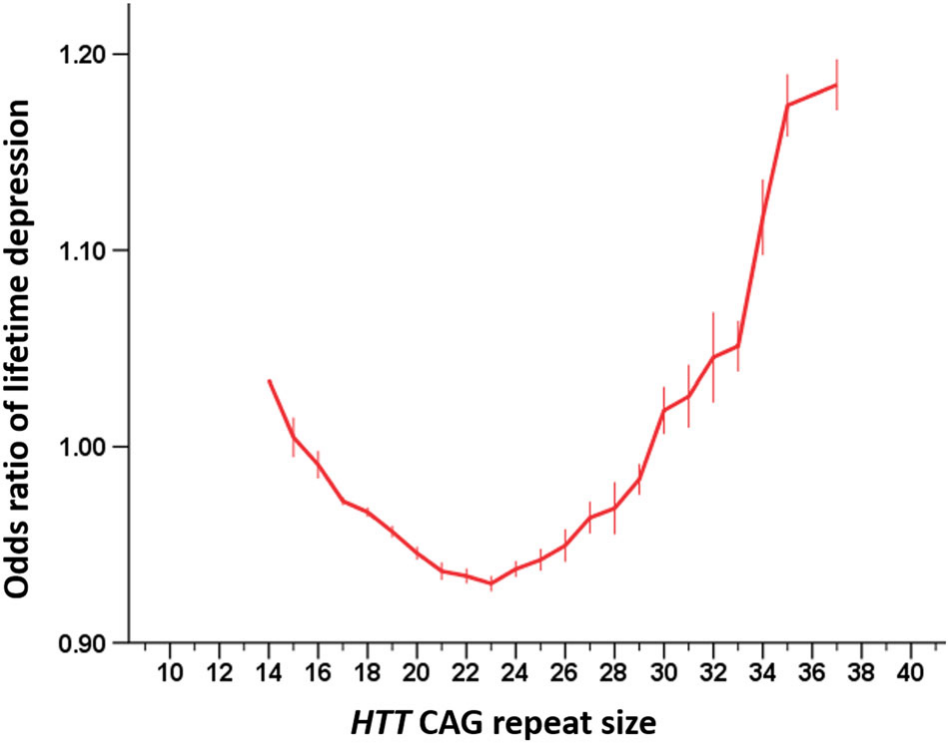
*HTT* CAG repeat size is non-linearly associated with lifetime depression risk The red line indicates the model predicted mean odds ratio for lifetime depression for each *HTT* CAG repeat size (adjusted for age, sex, and education level): Both subjects with a relatively low and a relatively high repeat size have a higher odds of developing depression compared to the odds of depression in the total group. Error bars indicate ± standard error. Please note that there was only one subject (with depression) who had a *HTT* CAG repeat size of 14 in the longer allele, therefore, no standard error could be calculated for this CAG repeat category. Please also note that the logistic model estimates of odds ratios for lifetime depression are slightly different from those presented in Table 1 as these estimates are obtained using data from all participants simultaneously, whereas the estimates in Table 1 are calculated for each subgroup of *HTT* CAG repeat size separately

**Fig. 3:**
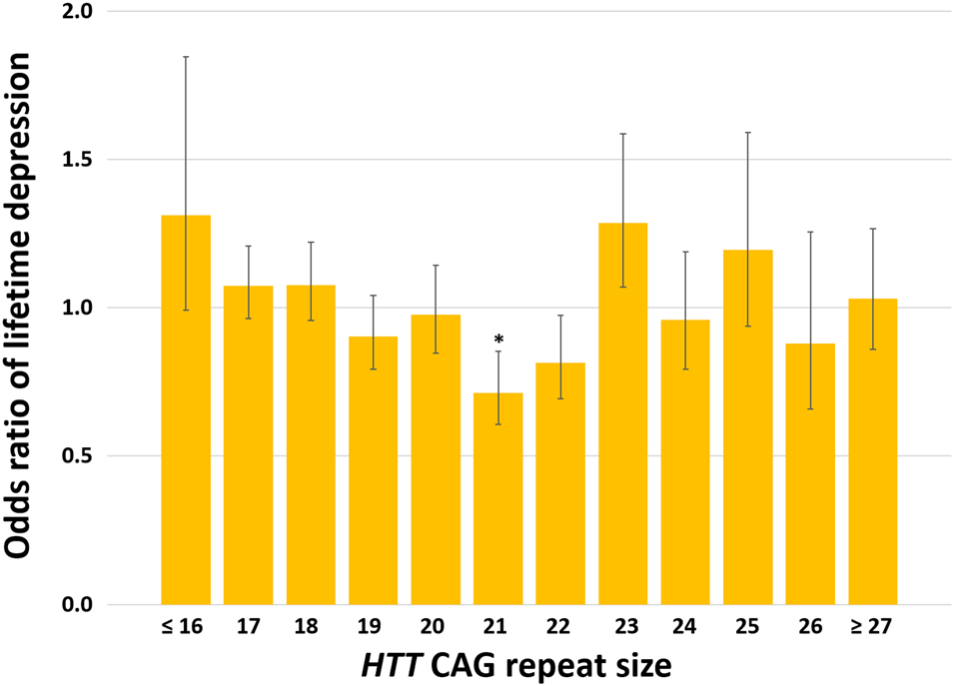
*HTT* CAG repeat size influences depression risk The odds of a diagnosis of lifetime depression is non-linearly associated with CAG repeat size in the longer *HTT* allele: Both subjects with a relatively low and a relatively high repeat size have a higher risk of developing depression compared to the odds of depression in the total group. Error bars indicate ± standard error. *P < 0.05 by the Fisher’s exact test in comparison to the odds of lifetime depression in the total cohort

Combining individual data from all subjects from both cohorts further increased the statistical significance of the results (*β* = −0.292 and *β* = 0.006 for the linear and the quadratic term, respectively; both *P* ≤ 0.009) after adjustment for the effects of sex, age, and education level, indicating that the effect of *HTT* on lifetime depression is independent of these risk factors (Fig. 2). Additional adjustment for cohort (i.e., NESDA or NESDO) hardly changed the statistical significance of the findings (*P* ≤ 0.008 for the linear and the quadratic term associated with *HTT* CAG repeat size). To evaluate whether the effect of *HTT* CAG repeat size is different for men and women, we also tested for the interaction between sex and *HTT* CAG repeat size. However, there was not a statistically significant interaction effect between sex and either the linear term (*P* = 0.077) or the quadratic term (*P* = 0.069) associated with *HTT* CAG repeat size, indicating that the effect of *HTT* CAG repeat size on depression susceptibility is the same for both men and women. Since our control group included people with anxiety disorders without co-morbid depression, we also performed a sensitivity analysis by excluding these subjects from the control group. This procedure did not materially change the parameter estimates or their statistical significance (*β* = −0.363 and *β* = 0.008 for the linear and the quadratic term, respectively; both *P* ≤ 0.006). Comparing the odds ratios of lifetime depression per *HTT* CAG category corroborated the results from the regression analysis and indicated that the risk of lifetime depression is lowest in individuals with a *HTT* CAG repeat size around 21 CAG repeats and increases with *both lower and higher* sizes of the CAG tract in this gene (Table 1 and Fig. 3). The Nagelkerke *R* ^2^ associated with a model with only a linear and a quadratic term for *HTT* CAG repeat size was 0.003, indicating that *HTT* CAG repeat size variations can account for 0.3% of the genetic variability of lifetime depression on the observed probability scale. We also derived the *R* ^2^ on the liability scale as described previously^31^. Assuming that depression has a lifetime prevalence of about 15% in the Netherlands^32^ and adjusting for the oversampling of patients with depression in our cohort, the *R* ^2^ on the liability scale was 0.004, indicating that *HTT* CAG repeat size polymorphisms can account for approximately 0.4% of depression heritability.

**Table 1:** Distribution of the CAG repeat sizes in the longer *HTT* allele

Repeat size	Lifetime depression^a^	No lifetime depression^b^	Odds ratio (95% confidence interval)^c^
≤ 16	51	19	1.31 (0.76 to 2.27)
17	532	242	1.07 (0.87 to 1.33)
18	414	188	1.08 (0.86 to 1.35)
19	257	139	0.90 (0.70 to 1.17)
20	208	104	0.98 (0.74 to 1.29)
21	121	83	0.71 (0.52 to 0.98)^*^
22	135	81	0.81 (0.59 to 1.12)
23	129	49	1.29 (0.90 to 1.85)
24	100	51	0.96 (0.66 to 1.39)
25	66	27	1.19 (0.74 to 1.92)
26	36	20	0.88 (0.50 to 1.55)
≥ 27	116	55	1.03 (0.72 to 1.47)
Total	2165	1058	

^a^Figures represent the number of subjects per *HTT* category in the group with lifetime depression

^b^Figures represent the number of subjects per *HTT* category in the group with no lifetime depression

^c^The odds ratio was calculated by dividing the odds of lifetime depression for each CAG repeat category by the odds of lifetime depression in the total cohort

^*^ *P* < 0.05 by the Fisher’s exact test in comparison to the reference category

Calculation of odds ratios based on data from cohort 3 confirmed a non-linear association between the risk of MDD and the number of CAG repeats in the *HTT* gene (Supplementary Fig. 1). However, because the data were reported on a chromosome level, and not on an individual level^24^, it was not possible to assess the effect of the shorter and the longer *HTT* allele separately in this cohort. In addition, because we could not reliably extract the frequency of individual allele categories with relatively rare repeat sizes of < 16 or > 27 from the article describing this cohort, we unfortunately could not perform a regression analysis. For comparison, however, we also analyzed our own data on chromosome level (i.e., comparing 4330 and 2116 chromosomes from patients with depression and controls, respectively): This analysis again confirmed a quadratic association between *HTT* CAG repeat size and risk of lifetime depression (*β* = −0.136 and *β* = 0.003 for the linear and the quadratic term, respectively; both *P* ≤ 0.023).

### The prevalence of intermediate and incompletely penetrant *HTT* alleles

In the combined cohorts 1 and 2, two individuals in the group with lifetime depression had a *HTT* CAG repeat size in the incompletely penetrant range (both with 37 repeats): one diagnosed with MDD and the other with dysthymia. HD was, however, not diagnosed at the time of assessment in either of them at the ages of 57 and 58 years, respectively. None of the subjects in the comparison group had an *HTT* CAG repeat size in the mutant range. The proportion of alleles with 27 CAG repeats or larger was 0.053 (95% confidence interval: 0.045 to 0.061) and did not differ between participants with depression and those without (*P* = 0.933).

## Discussion

To the best of our knowledge this is the first account of an association between CAG repeat size variations in the normal range of the *HTT* gene and risk of lifetime depression. Although previously associations have been reported between *HTT* CAG repeat size variations and depression risk, these studies focused either on the intermediate (i.e., 27–35 CAGs) or the incompletely penetrant (i.e., 36–39 CAGs) range^21–24^. Importantly, we found a non-linear relationship between CAG repeat size in the longer *HTT* allele and risk of lifetime depression in which *both relatively short and relatively large* alleles were associated with an increased risk of depression as compared to alleles containing around 21 to 24 repeats. Furthermore, in accord with a previous study we found *HTT* alleles in the incomplete penetrance range only in patients with depression while these were absent in the comparison group suggesting that some cases with depression in the general population might possibly be due to incipient HD^24^.

The presence of a curvilinear association between *HTT* CAG repeat size in the normal range and depression risk is intriguing as it suggests an optimal range of CAG repeat sizes in the *HTT* gene supporting the idea that polymorphic tandem repeats could act as genetic ‘‘tuning knobs’’ for evolutionary processes^33^. It is well-known that very long CAG repeat expansions in the *HTT* gene are associated with HD, which is accompanied by substantial neuropsychiatric disturbances of which depression is one of the most prevalent^12,13^. However, to date no disorders have been formally associated with very short CAG repeat tracts in the *HTT* gene. Although deletion of a terminal band on the short arm of chromosome 4, the region which contains the *HTT* gene, results in a distinct neurodevelopmental disorder known as the Wolf-Hirschorn syndrome and has been instrumental in localizing the *HTT* gene, this deletion concerns multiple genes and has not been associated with interruptions of the CAG repeat tract in the *HTT* gene^34^. In accord with our findings most population studies have found a minimum number of nine CAG repeats in exon 1 of *HTT* ^24,35–37^, suggesting selection against alleles with very small number of repeats. In this regard, it is also noteworthy that there is a ‘‘cliff’’ in the frequency distribution of *HTT* CAG repeat sizes between 16 and 17 repeats in our cohorts (Fig. 1), indicating that *HTT* alleles with less than 17 repeats are much less prevalent and, therefore, might be associated with a survival disadvantage. Moreover, a recent study also found that chromosomes from patients with bipolar disorder contained *HTT* alleles with significantly shorter CAG tracts than control chromosomes^36^. Although our cohort did not include patients with bipolar disorder, these findings are well in line with our results and suggest that both relative extremes in short and long *HTT* CAG tracts within the normal range could predispose to mood disturbances. Moreover, while depression is very prevalent among HD mutation carriers^38^, the relation between *HTT* CAG repeat size variations in either the expanded or the normal range with depression risk has not been systematically investigated before in HD patients or premanifest mutation carriers. Our findings, therefore, suggest that it would also be interesting to investigate the role *HTT* CAG repeat size variations as potential modifiers of depression in HD mutation carriers.

We can only speculate about the mechanisms that could account for the association between *HTT* CAG repeat size variations and risk of depression. Huntingtin is essential for normal embryonic development as targeted disruption of its encoding gene is lethal in mice^39^. Although huntingtin is expressed in many organs, its expression is highest in the brain^40^. Huntingtin is in fact critical for the development of the central nervous system since decreased levels lead to reduced neurogenesis and profound malformations of the cortex and striatum^40^. As the expression of huntingtin is modulated by its polyglutamine tract^40,41^, the effect of *HTT* CAG repeat size variations on depression might be due to altered endogenous levels of huntingtin. Importantly, huntingtin is a key regulator of brain-derived neurotrophic factor (BDNF)^41^. Reduced levels of BDNF have been consistently associated with depression, whereas normalization of BDNF levels has been reported following antidepressant treatment^42^. However, we did not have data on blood or cerebrospinal fluid levels of BDNF in our participants, therefore, whether small variations in *HTT* CAG repeat size in the normal range could affect *HTT* or *BDNF* gene expression remains to be investigated. Another mechanism that might account for the association between *HTT* CAG repeat size variations and risk of depression is the polyglutamine-length-dependent interaction of huntingtin with a large number of other proteins^40,43^, including several key transcription factors and co-activators such as cAMP response element-binding protein (CREB) and CREB-binding protein. These proteins are crucial for neuronal function and synaptic plasticity, disturbances of which have been implicated in depression pathogenesis^44^. Interestingly, mice with a targeted deletion of the CAG repeat tract of the HD gene exhibited defects in learning and memory, further suggesting an important role for the polyglumatine tract of huntingtin in central nervous system function^45^. Finally, altered regulation of the hypothalamic-pituitary-adrenal axis, which has been involved in the pathogenesis of both HD and major depression might play a role^46–50^. Although the precise cellular pathways through which *HTT* CAG repeat polymorphisms could affect mood remain to be elucidated, there are recent preliminary data showing an association between *HTT* CAG repeat size variations across the normal range and brain structure in healthy adults and children^51,52^, suggesting direct structural modulation of the brain as an underlying mechanism.

There are certain limitations to our study. First, a potential limitation of our study is that we only used samples from two Dutch cohorts in our main analyses. Although by analyzing a homogenous population we minimized the impact of population stratification, this might have consequences for the generalizability of our findings. However, analysis of data extracted from a previous publication based on samples from American participants confirmed our findings and suggests that the association between *HTT* CAG repeat size and depression susceptibility is also likely to exist in other populations. Second, there was a large difference in the age distribution between the two cohorts in our study (i.e., 18–65 and 60–93 years in the NESDA and NESDO cohort, respectively). It is of course possible that some of the younger individuals who have not suffered from depression yet will go on to develop depression in the future. However, it is unlikely that this might have affected our findings as we found the same non-linear association between *HTT* CAG repeat size and the odds of depression in both cohorts. Moreover, the results remained similar when we adjusted for age in the combined cohort. Therefore, our findings indicate that the association between *HTT* CAG repeat size and depression is independent of age. Third, although we did not have long-term follow-up data, it would be interesting to follow-up participants with *HTT* alleles in the incomplete penetrance range to assess whether, and when, they go on to develop the full spectrum of HD signs and symptoms. And finally, given our relatively limited sample size, our primary analysis was based on the results of the binary logistic regression including all the data available, however, fluctuating odds ratios per CAG repeat size (Table 1 and Fig. 3) might reflect a more complex relationship, which warrants further evaluation in larger cohorts in the future.

In conclusion, we found a non-linear association between CAG repeat size in the *HTT* gene and risk of lifetime depression in which lifetime depression risk increased with both relatively short and relatively large alleles compared to alleles with CAG repeat sizes near the center of the distribution. Our study provides important proof-of-principle that repeat polymorphisms could act as complex genetic modifiers of depression and thus may at least partly account for its ‘‘missing heritability’’.

## Electronic supplementary material

Supplementary Figure 1
