# Supplementary figures and images for: Huntingtin gene repeat size variations affect risk of lifetime depression
Source: Transl Psychiatry. 2017 Dec 11;7:1277. doi: 10.1038/s41398-017-0042-1 (PMC5802693; doi:10.1038/s41398-017-0042-1)

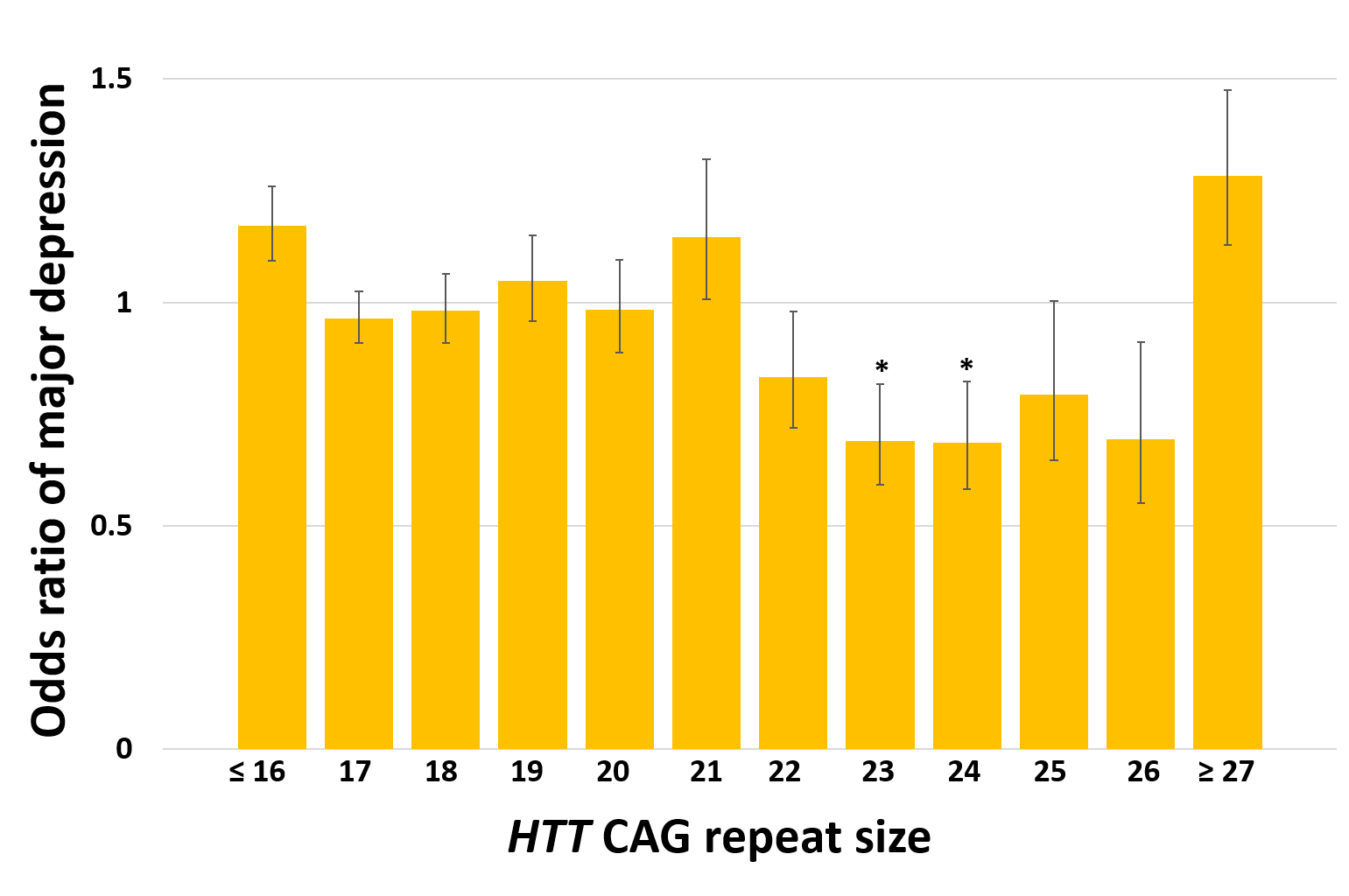

Supplement: Supplementary file 1 — Supplementary Figure 1 [file 41398_2017_42_MOESM1_ESM.tif]
